# Performance of Health Care Workers in Doffing of Personal Protective Equipment Using Real-Time Remote Audio-Visual Doffing Surveillance System: Its Implications for Bio-Safety Amid COVID-19 Pandemic

**DOI:** 10.7759/cureus.18071

**Published:** 2021-09-18

**Authors:** Naveen Naik B, Ajay Singh, Michelle S Lazar, Venkata Ganesh, Shiv L Soni, Manisha Biswal, Karobi Das, Sukhpal Kaur, Goverdhan Puri

**Affiliations:** 1 Department of Anaesthesiology, Postgraduate Institute of Medical Education and Research, Chandigarh, IND; 2 Department of Medical Microbiology, Postgraduate Institute of Medical Education and Research, Chandigarh, IND; 3 Department of Nursing Education, Postgraduate Institute of Medical Education and Research, Chandigarh, IND

**Keywords:** remote, doffing, covid-19, contamination, biosafety, breach

## Abstract

Background

Very little has been reported about health care workers' (HCWs) adherence to the Centers for Disease Control and Prevention (CDC) guidelines of doffing personal protective equipment (PPE) amid the COVID-19 pandemic. Real-time remote audio-visual doffing surveillance (RADS) system for assisting doffing might reduce the risk of self-contamination. We used this system to determine the incidence of the breach in biosafety during doffing of PPE among HCWs involved in the care of Covid-19 patients.

Methods

A total of 100 HCWs were enrolled in this observational study who performed duties in the COVID intensive care unit (ICU) of our tertiary care centre. With a real-time RADS system, trained observers from remote locations assisted HCWs during doffing of PPE and noted breach at any step using the CDC doffing checklist. The breach was considered major if committed during removal of gloves/gown/N-95 or if ≥3 errors occurred in any other steps.

Results

Overall, 40% of the HCWs committed a breach during doffing at least one step. The majority of the errors were observed during hand hygiene (34%), followed by glove removal (12%) and N-95 removal (8%). Nineteen percent of HCWs committed the major breach, out of which 37.5% were done by house-keeping sanitation staff (p = 0.008 and RR 2.85; 95% CI of 1.313-6.19), followed by technicians (22.5%), nursing staff (16.7%) and resident doctors (6.5%).

Conclusions

Performing doffing using a real-time RADS system is associated with a relatively low incidence of a breach in biosafety compared with earlier studies using an onsite standard observer. Overall adherence of HCWs to the CDC guidelines of doffing PPE was satisfactory. This study highlights the importance of the RADS system during doffing of PPE in a health care setting amid the COVID-19 pandemic.

## Introduction

Prompted by the menace of Coronavirus disease 2019 (COVID-19) and its implications all over the world, the Centers for Disease Control and Prevention (CDC) augmented efforts to provide safe care for patients with suspected or confirmed COVID-19 [1]. COVID-19 endangers the health of all, but especially that of the health care workers (HCWs) involved in patient care. Personal protective equipment (PPE) protects HCW from the risk of exposure to COVID-19 and enables them to deliver safe and effective patient care [2]. PPEs suggested by the CDC comprise N95 mask, eye protection, gloves, and gowns [3]. Incorrect doffing of PPE by HCWs could potentially cause a breach in bio-safety and lead to self-contamination [4]. At present, the CDC recommends proper sequences for donning and doffing of PPE and safe practices to limit the spread of contamination [5]. Reinforcing them during training in PPE use can hone technical skills and reduce self-contamination risk among HCWs while doffing. Observational studies have shown that lapses do happen while doffing [6,7] and lead to self-contamination, even though HCWs presume they are competent in doffing of PPE [8]. Literature suggests higher self-contamination rates of 46%-100% among HCWs while doffing of PPE [9-12].

During doffing, HCWs frequently self-contaminate while taking off gloves [12,13], gowns [7,14], respirator and hood [15,16]. Other contributing factors include incorrect doffing sequences, difficulty in distinguishing between dirty and clean surfaces, rushed movements [17,18] and suboptimal PPE training. Additional interventions beyond training in PPE use may be necessary to limit deviations from the standard protocol further. Surveillance [19], simulation-based training and assisted doffing, strictly following the checklists can minimize the cognitive load among HCWs and increase performance while doffing PPE amid the current COVID-19 pandemic.

The Healthcare system should function at the highest standards, utilizing the best available technology and resources for better patient care, staff safety and communication. A health care setting with multiple doffing areas usually requires varying levels of assistance. In such a setting, ensuring HCW adherence to PPE doffing protocol with the help of onsite standard observers is laborious and may not be feasible always. Taking advantage of simple technologies leveraged to ensure the safety of frontline HCWs during doffing is pivotal. Technology should comprise a video surveillance system integrated with a communication platform so that necessary stakeholders can be instantly apprised.

For the reasons stated above, we implemented real-time remote audio-visual doffing surveillance (RADS) system utilizing high-definition closed-circuit television (CCTV) surveillance cameras installed in several doffing areas to remotely monitor and assist the HCW in doffing PPE [20]. In the present study, we aimed to observe a breach in biosafety among different HCWs during doffing of PPE using a real-time RADS system. The types and frequencies of the breach in biosafety observed in the CDC doffing sequence were also determined.

## Materials and methods

This prospective observational study was conducted at the COVID block of a tertiary care institute in the northern part of India. The study was approved by the Institutional Ethics Committee PGIMER Chandigarh and registered in clinical trials registry India (CTRI), with reference number CTRI/2020/05/025274. This study was conducted between June 1, 2020, and July 3, 2020.

A total of 100 HCWs were enrolled for the study. Participation was voluntary. HCWs were informed about the characteristics and scope of the study. All the participants signed an informed consent form. Study participants were all members of the COVID-19 care team of our institute. They were involved in the care of confirmed COVID-19 patients in ICU. As per our hospital policy, all the participants had undergone mandatory training in PPE use, including donning and doffing practices based on CDC recommendations, before performing duties in COVID ICU. The training was given according to the CDC guidelines at the time by trained faculty. This training was mandatory for every novice health care worker and was done under direct supervision. The training took place over a period of seven days before the health care worker was posted in their respective work area. HCWs were excluded as team members if they were pregnant, immuno-compromised or had inflammatory skin conditions. HCWs who refused to participate as well as the investigators in this study, were excluded.

**Figure 1:**
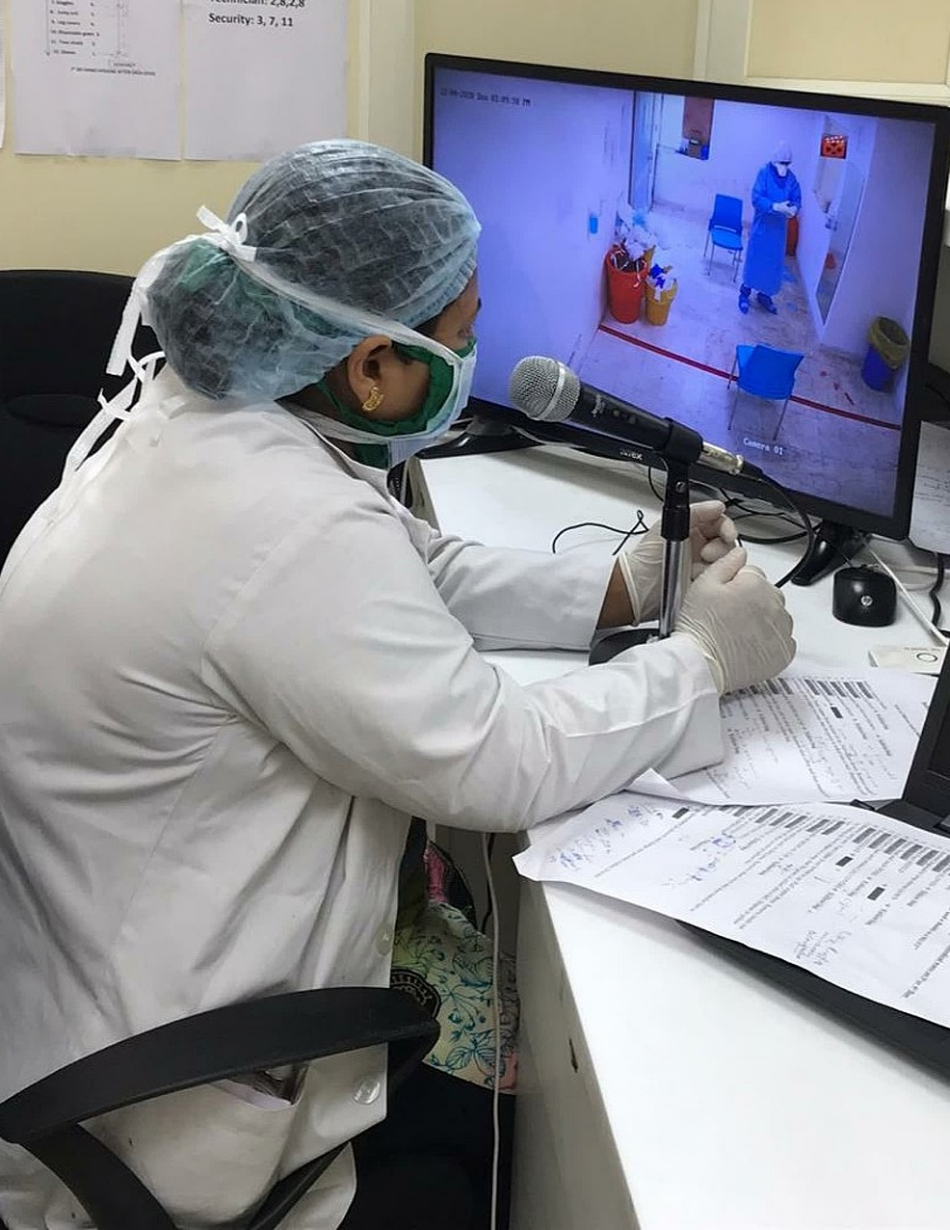
A trained observer assisting doffing process from a remote location using a real-time RADS system. RADS: Remote Audio-visual Doffing Surveillance

The doffing process was visualized remotely in a console room utilizing CCTV cameras installed in the doffing area and verbally communicated using the audio platform (Figure 1). A doffing checklist was developed based on the CDC recommendations (Appendix 1) and was used by trained observers for ensuring HCW adherence to doffing sequence [21]. Using this audio-visual communication system, a trained observer from the console room guided the HCWs throughout the doffing process, marking the CDC doffing checklist. The trained observers were registered nurses and certified infection preventionist, who monitored and assisted the HCW doffing of PPE round the clock. A silent observer (intensivist) in the console room who was not a part of this study monitored the doffing process of the HCW and noted any breach or error in biosafety at any step in the doffing checklist.

A major breach in the doffing process was considered if the HCW committed any error while removing (1) outer gown, (2) gloves (both outer and inner pair), (3) N-95 mask, or (4) if an error occurred at least three or more times during the remaining steps of CDC doffing sequence. If there was no breach in biosafety at any step, then the doffing process was considered error/breach-free. If any HCW committed a major breach in biosafety while doffing PPE, it was notified to the concerned authorities as per the hospital protocol.

Statistical analysis

As the previously published studies on doffing of PPE in a clinical setting are limited by the number, fewer data and the types of data analyses, no formal sample size calculation was done in this observational study. We intended to include a minimum of 100 volunteers, by convenience sampling, working in the designated COVID ICU during the study period. Univariate analyses were performed, and the p-value of ≤ 0.05 was considered significant. We used χ2 (chi-square) for categorical variables. Relative risks were calculated, and descriptive data has been presented. Analyses were performed with SPSS version 25 (IBM Corp., Armonk, NY).

## Results

In total, 100 HCWs were observed during the doffing of PPE through a real-time RADS system. Of these participants enrolled in the study, 31% (n = 31) were resident doctors, 36% (n = 36) were nursing staff, 24% (n = 24) were housekeeping sanitation staff, and 9% (n = 9) were technicians. The demographic parameters and shift timings have been detailed in Table 1.

**Table 1:** Healthcare worker demographics ^a^ Unless otherwise specified HCW: Health care worker; PPE: Personal protective equipment.

Characteristics	HCW (n = 100) Number (%)^ a^
Age in years, median (range)	32 (20-48)
Male/Female (number)	52/48
HCW type	
Resident Doctor	31 (31)
Nursing staff	36 (36)
Environmental sanitation worker	24 (24)
Technician	9 (9)
Previous PPE training received	100 (100)
Duty Shift	
8 am-2 pm	25 (25)
2 pm-8 pm	29 (29)
8 pm-2 am	19 (19)
2 am-8 am	27 (27)

The majority of the HCWs (60%) did not commit breach in biosafety in any step of CDC doffing protocol, while 40% of them deviated from protocol in at least one step. The major deviation occurred during hand hygiene (multiple steps) (34%), removal of outer and inner gloves (12%) and while N-95 removal (8%). Protocol deviation in 1-2 steps was committed by 30% HCWs, in 3-4 steps by 7% HCWs and only 3% HCWs deviated from protocol in more than four steps.

The major breach in biosafety was committed by 19% of HCWs, out of which 37.5% (i.e., nine of 24) were sanitation staff, followed by 22.5% technicians (two of nine) and 16.7% (six of 36) nursing staff. Only 6.5% of doctors (2/31) committed a major breach in biosafety. The probability of sanitation workers committing a major breach was 2.85 times that of other HCWs (χ2, P-value = 0.008 and RR 2.85; 95% CI of 1.313-6.19). 19.6% of the HCWs in the night shifts (8 pm to 8 am) and 18.5% of those in day shifts (8 am to 8 pm) committed a major breach. The distribution of major breaches was equal during the night (47.37%, 8 pm to 8 am) and day (52.63%, 8 am to 8 pm) duty shifts. Those who worked in the night duty shifts had a relative risk of only 1.057 (95% CI of 0.470-2.375) of committing a major breach compared to those working in the day duty shifts (Table 2).

**Table 2:** Characteristics of deviations in CDC doffing protocol among HCWs ^a ^Major breach/error is defined as an error committed while removal of either gown, gloves, N95 respirator or more than three errors in any other step. HCW: Health care worker

Characteristics	HCW, Number (%)
Error/Deviation during doffing	
Any step	40 (40)
Hand hygiene (multiple-step)	34 (34)
Removal of outer and inner gloves	12 (12)
Removal of N95 mask	8 (8)
Removal of Apron/gown	2 (2)
Removal of Other PPE components (Face Shield, Shoe cover, leg cover, etc.)	17 (17)
Number of HCW deviating from the protocol	
No deviation	60 (60)
Deviation in 1-2 steps	30 (30)
Deviation in 3-4 steps	7 (7)
Deviation in more than 4 steps	3 (3)
HCWs who made major breach/error^a^	19 (19)
Type of HCWs committing major errors	
Resident doctor (n = 31)	2 (6.5)
Nursing staff (n = 36)	6 (16.7)
Environmental sanitation worker (n = 24)	9 (37.5)
Technician (n = 9)	2 (22.2)
Duty shift timings and number of HCWs committing major errors	
8 am-2 pm (n = 25)	5 (20)
2 pm-8 pm (n = 29)	5 (17.2)
8 pm-2 am (n = 19)	6 (31.6)
2 am-8 am (n = 27)	3 (11.1)

Forty HCWs made a total number of 78 errors. Breach in biosafety at various steps of CDC doffing protocol by different HCWs is shown in Table 3. None of the trained observers in the console room committed protocol violations during the study period while assisting HCWs in doffing PPE. And as per our hospital policy, none tested positive when all the HCWs were tested with real-time reverse transcriptase-polymerase chain reaction (RT-PCR) to detect COVID-19.

**Table 3:** Distribution of errors at each step of CDC guidelines of doffing among different HCWs. *Considered as major step and error/deviation in any of these steps was considered significant. ^a ^Chi-square test; ^b ^P < 0.05 is considered significant

SI No	CDC doffing protocol steps	Resident Doctor (n = 31)	Nursing staff (n = 36)	Housekeeping sanitation staff (n = 24)	Technician (n = 9)	No. of HCWs who made error/deviation (n = 100)	P-value^a^
1	Inspect	0	0	0	0	0	---
2	Disinfect Outer Gloves	1	3	6	4	14	.004^b^
3	*Remove Apron	0	0	2	0	2	.091
4	Inspect	0	0	0	0	0	---
5	*Disinfect and Remove Outer Gloves	1	2	3	1	7	.543
6	Inspect and Disinfect Inner Gloves	0	0	3	0	3	.020
7	Remove Face Shield	0	1	1	3	5	.001^b^
8	Disinfect Inner Gloves	0	1	1	0	2	.682
9	Remove Surgical Hood	0	1	0	0	1	.616
10	Disinfect Inner Gloves	0	0	0	0	0	
11	Remove Hazmat or Coverall	4	1	2	2	9	.239
12	Disinfect Inner Gloves	0	0	1	0	1	.362
13	Remove Boot Cover	1	4	0	0	5	.189
14	Disinfect and Change Inner Gloves	0	2	0	1	3	.208
15	*Remove N95 Respirator	1	3	4	0	8	.241
16	Disinfect Inner Gloves	1	0	1	0	2	.630
17	Disinfect Washable Shoe	0	0	0	0	0	
18	*Disinfect and Remove Inner Gloves	0	1	4	0	5	.025
19	Perform Hand Hygiene	1	2	4	2	9	.143
20	Inspect	0	1	0	0	1	.616
21	Scrubs	0	0	1	0	1	.362
	Total number of errors (%)	10 (12.8)	22 (28.2)	33 (42.3)	13 (16.6)	78	

## Discussion

The ongoing COVID-19 pandemic has had a significant impact on global healthcare services. India has the third-highest number of confirmed cases globally after the United States and Brazil, with more than 1.2 million total confirmed COVID-19 cases and more than 30,000 total deaths, as of July 23, 2020 [22]. Government strategies and population responses have not resulted in flattening the epidemic curve, which implies an expected spread over a longer period, for many months or even years. For the frontline HCWs involved in the patient care, the greatest risk factors for getting infected with COVID-19 is (1) exposure from an infected patient during care and (2) self-contamination during the doffing of PPE [4]. The importance of preventing exposure to SARS-CoV-2 during patient care cannot be understated, as more than 90,000 HCWs have developed COVID-19 worldwide as of May 7, 2020 [23]. There is no central registry of confirmed cases of HCWs existing in India; however, New Delhi alone has reported more than 2000 infected HCWs, as of June 20, 2020 [24].

The appropriate use of PPE is essential to reduce the number of infected healthcare workers caring for patients with COVID-19 [4]. The most overlooked aspect of the alarming COVID-19 case numbers is whether HCW is doffing PPE properly without self-contamination. Osei-Bonsu et al. reported a 90% self-contamination rate among HCWs during doffing of PPE, underlining the necessity for paying close attention to the doffing technique [11]. Kang et al. estimated that self-contamination occurred in 79.2% of simulations and 66% of HCWs while doffing PPE after caring for patients in isolation precautions. They highlighted the need for devising innovative methods to ensure HCW safety during doffing [9]. Kwon et al. reported 100% self-contamination among all HCWs doffing PPE used for Ebola patient care, where all the HCWs made ≥1 protocol deviation while doffing [10]. The effective practices of donning and doffing PPE are essential for COVID-19 prevention in a health care setting. WHO recommendations prioritize the significance of correct usage of PPE, which necessitates meticulous behaviour from health care workers, especially during doffing [25]. Beam et al. demonstrated that simple exposure to a poster showing the correct doffing sequence might not be sufficient [26]. A breach in biosafety can occur at any step in the sequence of doffing. Despite their knowledge of PPE use, HCWs do not carry out an appropriate donning and doffing process. Díaz-Guio et al., based on their simulation study, recommended assisted donning and doffing amid the COVID-19 pandemic [27]. Kwon et al. reported 15 protocol deviations committed by the onsite trained observers while assisting the doffing of PPE [10]. Findings from our study suggest that utilizing a doffing checklist with audio-visual communication might reduce the protocol deviations committed by trained observers.

Experiencing the formidable outlook of the COVID-19 pandemic with a deluge of sick patients getting admitted to ICUs, HCWs responsible for their care are often busy and doff PPE frequently. Providing round-the-clock assistance to them while doffing PPE is of utmost importance. Attempts to meliorate doffing of PPE should include both prudence and safety, utilizing innovative or improved methods, training practices, and organizational policies [28]. An imminent crisis is opportunities for innovation, wherein a conventionally slow-moving healthcare facility can be improvised in response to the pandemic [29]. To stem the risk of self-contamination during doffing of PPE, we utilized the real-time RADS system to assist HCW while doffing [20]. With this system, a trained observer from an offsite location can easily collaborate through a visual screen, imparting crystal-clear communication while assisting doffing. The immediacy and ease of this system are crucial in guiding HCW during doffing PPE.

**Figure 2:**
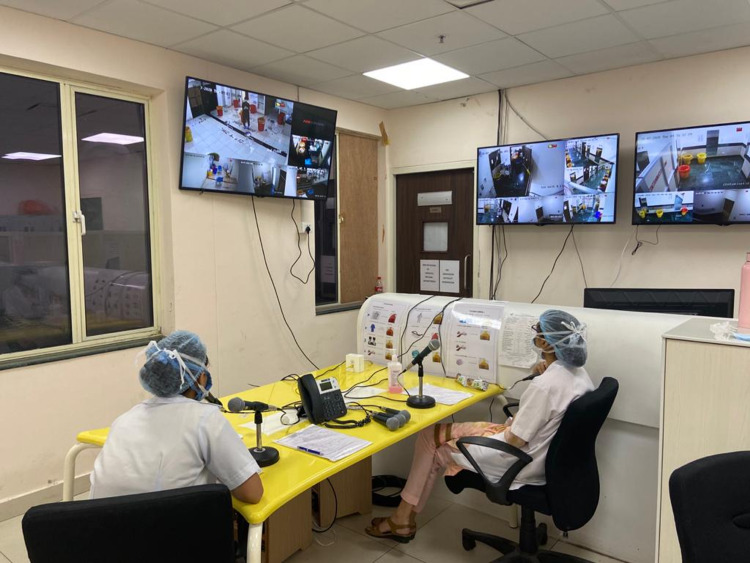
Console room with trained observers assisting doffing process in multiple doffing areas with real-time RADS system. RADS: Remote audio-visual doffing surveillance

With the growing burden on the hospital with increasing ICU units to care for COVID-19 patients on a large scale, hospitals tend to increase the number of designated doffing areas. With these areas generally spanning several buildings or locations, the integration potentiality of such simple and unified audio-visual technologies is essential. The real-time RADS system is convenient and can bring great value for HCWs doffing in many doffing areas simultaneously (Figure 2). The flexibility of such a platform allows the observer to communicate multiple messages with ease. For example, with multiple doffing areas requiring varying access levels, this system has the added advantage of ensuring the disposal of used PPEs from these areas. With PPE shortage posing a major challenge to the healthcare facilities in the ongoing pandemic, CDC has recommended conservation strategies for optimizing its use [1]. PPE use by on-site trained observers in different duty shifts for assisting doffing can be minimized with this novel surveillance system. And also, by using this system, a calm off-site observer may be better able to guide the doffing process than the exposure-prone anxious onsite observer.

To our knowledge, this is the first study to use the real-time RADS system to inspect and assist HCW in doffing PPE and monitor their adherence to CDC doffing protocol. Our organisation operating this system so far has already begun perceiving the benefits. Overall, 40% of HCWs committed a breach in biosafety at any step in the doffing protocol, and 19% made a major breach in biosafety in our study. Kwon et al. reported 100% incidence in the breach at any step among HCWs during doffing PPE with the help of an onsite standard observer [10]. Okamoto et al. reported a 39.2% incidence of multiple doffing errors despite prior training [30]. Errors during hand hygiene and removal of gloves/gown/N-95 were the most common during the doffing of PPE [10,17]. Our study demonstrated reduced incidence rates of breaches during hand hygiene (34%), removal of gloves (12%), N-95 removal (8%) and gown/apron removal (2%). Previous studies have observed violations in doffing protocol at either one step or multiple steps in simulated environments using surrogate markers like fluorescent materials and/or bacteriophages [9-12]. The findings of our study supplement these observations to the actual world of a tireless clinical setting where it is probable that HCWs could deviate from PPE doffing protocols; nevertheless, they have received training [18]. However, despite committing errors while doffing, none of our HCWs had developed any symptoms of COVID-19 or were tested positive with RT-PCR testing at the end of seven-day post-duty sampling.

Our study has the following limitations. First, the data of previous simulation studies using surrogate markers may not be comparable to our observational study performed in a clinical setting. Second, swab samples from PPE of HCWs during/after doffing were not collected for RT-PCR testing to detect/confirm the presence of the virus, and the only deviation from protocol was observed. However, all the tested PPE swab samples may not always be positive, suggesting all breaches in biosafety may not lead to self-contamination. Further studies are required to confirm our preliminary results. Third, our results may not be reflective of HCW populations at large.

Based on our observations, we speculate that performing doffing using a real-time RADS system is associated with a low incidence of the breach in biosafety and decreased protocol deviations compared with doffing assistance using onsite standard observers or posters in the doffing area. Utilizing this surveillance system to assist doffing can replace indistinct or inconsistent practices with trouble-free intercommunications that almost replicate real-life face-to-face meetings. The ability to guide the HCW throughout the process of doffing can reduce their anxiety levels and provide a pleasant experience overall. Systems like real-time RADS should be widely implemented to reduce healthcare-associated COVID-19 in HCWs. Our methods and results lay the foundation for future research in a larger population. The current pandemic is a tremendous opportunity for health care planners to strengthen the existing health systems or search for innovative methods to ensure the safety of HCWs caring for COVID-19 patients.

## Conclusions

The ongoing COVID-19 pandemic poses a remarkable burden on the health care system. Appropriate doffing of PPE remains crucial to decrease the infection rate among healthcare workers. With several HCWs requiring assistance while doffing PPE in multiple doffing areas simultaneously, trained observers can coordinate with them efficiently round the clock using a real-time RADS system. This system helps by reducing the requirement of a donned observer, thus conserving PPE and potentially reducing exposure to the observer while preserving the standard of safety while doffing. This study highlights the benefits of a real-time RADS system in lowering the probability of committing a breach in biosafety during doffing of PPE. Hence transforming into a lesser illness burden amongst HCWs involved in the care of COVID-19 patients, round the clock.

## Appendices

**Table 4:** CDC recommended steps of doffing protocol (checklist).

SI. No	Step	Correctly done	Error made
	Engage trained observer		
	Inspect		
	Disinfect outer gloves		
	Remove apron		
	Inspect		
	Disinfect and remove outer gloves		
	Inspect and disinfect inner gloves		
	Remove face shield		
	Disinfect inner gloves		
	Remove surgical hood		
	Disinfect inner gloves		
	Remove coverall		
	Disinfect inner gloves		
	Remove boot covers		
	Disinfect and change inner gloves		
	Remove N95 respirator		
	Disinfect inner gloves		
	Disinfect washable shoes		
	Disinfect and remove inner gloves		
	Perform hand hygiene		
	Inspect		
